# Invasive growth of *Aspergillus oryzae* in rice *koji* and increase of nuclear number

**DOI:** 10.1186/s40694-020-00099-9

**Published:** 2020-06-05

**Authors:** Mizuki Yasui, Ken Oda, Shunsuke Masuo, Shuji Hosoda, Takuya Katayama, Jun-ichi Maruyama, Naoki Takaya, Norio Takeshita

**Affiliations:** 1 Microbiology Research Center for Sustainability (MiCS), Faculty of Life and Environmental Sciences, University of Tsukuba, Tsukuba, Japan; 2 National Research Institute of Brewing, 3-7-1 Kagamiyama, Higashi-hiroshima, Hiroshima, 739-0046 Japan; 3 Department of Biotechnology, Collaborative Research Institute for Innovative Microbiology, The University of Tokyo, Tokyo, Japan

**Keywords:** *Aspergillus oryzae*, *koji*, Rice, Nuclei, Mitosis

## Abstract

**Background:**

‘Rice *koji*’ is a solid culture of *Aspergillus oryzae* on steamed rice grains. Multiple parallel fermentation, wherein saccharification of rice by *A. oryzae* and alcohol fermentation by the budding yeast occur simultaneously, leads to the formation of a variety of ingredients of Japanese sake. In sake brewing, the degree of mycelial invasive growth into the steamed rice, called ‘*haze*-*komi*’, highly correlates with the digestibility and quality of rice *koji*, since the hyphae growing into the rice secrete amylases and digest starch.

**Results:**

In this study, we investigated mycelial distribution of GFP-tagged *A. oryzae* in rice *koji* made with different types of rice, such as sake rice and eating rice, with 50 or 90% polishing rate to remove abundant proteins and lipids near the surface. In addition, we compared transcriptomes of *A. oryzae* in the different types of rice *koji*. Finally, we found that *A. oryzae* increases the nuclear number and hyphal width in the course of 1–3 days cultivation.

**Conclusions:**

Our imaging analyses indicate that *A. oryzae* hyphae grew more deeply into 50% polished rice than 90% polished rice. The increases of nuclear number may be a selectively acquired characteristic for the high secretory capacity during the long history of cultivation of this species.

## Introduction

Filamentous fungi secrete a variety of enzymes to degrade extracellular organic compounds, serving as decomposers in nature [1]. In addition, modern biotechnologies have utilized filamentous fungi as cell factories for the production of organic acids, drugs including antibiotics and enzymes due to high secretory capacity [2]. Fungal biotechnology plays a central role for many industries such as food and feed, pharma, detergent, bio-fuel and others. Filamentous fungi grow by hyphal tip growth, forming multi-cellular networks with branching cells at subapical regions [3–5]. Hyphae and mycelial networks are specifically adapted for growing on solid surfaces and invading substrates and tissues [6].

The filamentous fungus *Aspergillus oryzae* has been used in the production of traditional fermented foods such as sake (rice wine), miso (soybean paste) and shoyu (soy sauce) for more than 1000 years in Japan [7]. The safety of *A. oryzae* is guaranteed by the long history of use in food fermentation industries and molecular genomic and metabolomic analyses [8, 9], which is supported by the World Health Organization [10]. *A. oryzae* has been used commercially as a host for homologous and heterologous protein production as well in modern biotechnology [11, 12]. One of the distinctive features in the use of *A. oryzae* in Japanese traditional fermentation is the use of solid-state cultivation (rice grain, soybean and wheat bran). Since filamentous fungi often secrete more enzymes in solid-state culture than in submerged culture [13–15], several commercial enzymes are produced in solid-state culture in addition to traditional fermentation methods. For example, glucoamylase and acid protease encoding genes, *glaB* and *pepA*, respectively, are known as the solid-state-specific genes of *A. oryzae* [16–18]. Transcriptome and proteome analyses have revealed the ability of *A. oryzae* to produce heterologous proteins in solid-state culture [15, 19].

‘Rice *koji*’ is a solid culture of *A. oryzae* on steamed rice grains. Multiple parallel fermentation, wherein saccharification of rice by *A. oryzae* and alcohol fermentation by the budding yeast (*Saccharomyces cerevisiae*) occur simultaneously, leads to the formation of a variety of ingredients in Japanese sake with its characteristic tastes. Sake contains more than 280 metabolites that affect its quality. The metabolite composition of sake depends on the combination of raw materials and sake-making parameters (e.g., rice races, rice polishing ratio, water quality, *koji* mold, yeast strains, sake mash fermentation methods) used during manufacturing [20]. In sake brewing, the degree of hyphal penetration into the steamed rice, called ‘*haze*-*komi*’, highly correlates with the digestibility and quality of *koji* [21–25], since the hyphae growing into the rice secrete amylases and digest the starch. The *haze*-*komi* is believed empirically to determine the distribution and composition of enzymes in rice *koji*, the step of fermentation and the qualities of sake. Recently, the hyphal penetration into steamed rice was visualized by β-glucuronidase (GUS)-expressing *A. oryzae* [26]. The correlation with the spread of glucose during fermentation was shown by mass spectrometry imaging (MSI) of glucose [26].

Japanese people normally consume eating rice as food, while sake is made with sake rice, which is especially suitable to sake brewing. Features of sake rice are large grains generally with “white core” (or *shinpaku* in Japanese), a low protein content and high solubility during the brewing process, which facilitates *A. oryzae* invasive growth [27]. Distribution of nutrients in a grain of rice is not uniform. Proteins and lipids are abundant near the surface of rice, resulting in miscellaneous taste and off-flavor of sake [28]. The Japanese high-quality sake, *daiginjo*-*shu*, is made from highly polished rice with polishing ratio less than 50% [29].

In this study, we investigated mycelial distribution using GFP-tagged *A. oryzae* in rice *koji* made with different types of rice (sake rice or eating rice, 50 or 90% polishing ratio).

## Materials and methods

### Rice and fungal species

*Yamada*-*nishiki* (sake rice) and *Chiyo*-*nishiki* (eating rice) were used to make *koji*. The rice grains were polished to different ratios (50% and 90%) using a milling machine (HS-08CNC, Chiyoda, Hiroshima, Japan). 90% polishing rate means polishing 10% outside of rice. *Yamada*-*nishiki* and *Chiyo*-*nishiki* with polished rate 50% or 90% are referred as Y50, Y90, C90, respectively. The H2B ORF was amplified from the genomic DNA of *A. oryzae* wild strain RIB40 using the primers PamyB-H2BF: 5′-TCGAGCTCGGTACCCATGGCACCCAAGGCTGCTGA-3′, Tag-H2BR: 5′-CAAGAAAGCTGGGTCCCCTTTGGCAGAAGAGGAGTACTTCGTA-3′ and then ligated with the SmaI-digested plasmid pUtNAN [30], yielding the plasmid pUtH2BG. The NotI-digested pUtH2BG was introduced into the RIB40Δn strain [31], yielding the strain RIB40UtH2BG expressing H2B-GFP. *A. oryzae* strain RIB40UtH2BG (pUtH2BG) was used to make *koji*. *Aspergillus nidulans* strain SRS27 (*gpdA* promoter GFP fused StuA-NLS) [32] and *Neurospora crassa* N22813A strain [33] were used to monitor nuclei.

### Small-scale *koji* making

2–5 g of the rice were soaked in water until the weight increased 30%; Y50 for 6 min 40 s, Y90 and C90 for 120 min. They were steamed for 10 min in a steamer, with the lid of steamer remaining closed for 15 min after turning off the heat. The steamed rice was inoculated with spores of *A. oryzae*, 10^4^ spores per rice 1 g, and incubated in lab dishes with papers containing 4 ml water to keep humidity at 30 °C for 33 h. Temperature of the incubator was raised to 42 °C gradually for 15 h, total 48 h.

### Rice *koji* sectioning

One grain of rice *koji* was embedded with the optimal cutting temperature compound (Sakura-finetech, Japan) in a mold, and frozen at − 80 °C. The frozen sample block was sectioned by using a cryostat (CM1850, Leica microsystems). Longitudinal sections with 30 μm thickness were obtained from the approximate center of the rice *koji*. Adhesive film was used to acquire the sample sections. Chamber and sample holder temperatures were kept at -20 °C. The sections were placed on a glass slide for imaging analysis.

### Microscopes

The confocal laser scanning microscope (CLSM) LSM880 (Carl Zeiss, Jena, Germany) equipped with a 63×/0.9 numerical aperture Plan-Apochromat objective was used to acquire confocal microscopic images. We visualize fungal and rice cells by CLSM and confocal reflection microscopy. Fungal nuclei and rice cells were visualized with 488 and 400-nm lasers, respectively. Acquired confocal images were analyzed using ZEN Software (Version 3.5, Carl Zeiss) and ImageJ software. Another CLSM TCS SP5 (Leica, Mannheim, Germany) equipped with HC PL APO 20x/0.75 IMM CORR CS2 objective lens was used to acquire confocal dual-color microscopic images. For epi-fluorescent inverted microscopy, cells were observed using an Axio Observer Z1 (Carl Zeiss) microscope equipped with a Plan-Apochromat 63  ×  1.4 Oil or 10 or 20 times objective lens, an AxioCam 506 mono camera and Colibri.2 LED light (Carl Zeiss). Temperature of the stage was kept at 30 °C by a thermo-plate (TOKAI HIT, Japan). For zoom microscopy, plates were observed by AXIO Zoom V16 and HXP 200C illuminator (Carl Zeiss). Images were collected and analyzed using the Zen system (Carl Zeiss) and ImageJ software.

### SEM

Benchtop scanning electron microscope JCM-6000 (JEOL, Japan) was used to observe *koji* section cut by a scalpel in low vacuum condition without special treatment.

### Transparentizing of rice

We made rice tissues of rice koji transparent according to the attached protocol of tissue-clearing reagent TOMEI (Tokyo Chemical Industry, Japan). One grain of rice *koji* was soaked in 4% Paraformaldehyde in PBS (phosphate-buffered saline) at room temperature in the dark for 30 min. The buffer was replaced with PBS and incubated for 5 min, then washed three times by incubating for 10 min with new PBS. The rice *koji* was incubated in 10, 30, 50, 70, 100% TOMEI regent (2,2′-Thiodiethanol containing Propyl Gallate, PBS and DMSO) for 10 min. The sample in TOMEI regent was used for imaging analysis.

### X-ray computed tomography

Mycelia in the rice *koji* are detected by an X-ray CE, SMX-160CTS (Shimadzu). One grain of rice *koji* was placed on the rotary table between the X-ray tube and the X-ray detector, and rotated to collect X-ray data from all angles. The volumes of mycelia are calculated by VG Studio MAX software.

### RNA-seq analysis

To isolate the total RNA from fungal cells in rice *koji*, rice *koji* was frozen and homogenized using mortar and pestle, and then the total RNA was extracted using an RNA isolation kit (RNA Mini Kit, Zymo Biomics). Novogen Inc. conducted library preparation, sequencing and partial data analysis. Each sample is sequenced using 150-bp paired-end reads on an Illumina NovaSeq 6000 instrument. The reads are mapped to the reference genome of *A. oryzae* RIB40, NCIB ID: 510516, through CLC Genomic Workbench (QIAGEN). After log2 transformation of RPKM + 1 and quantile normalization, differentially expressed genes were selected on conditions of log2 > 2 in expression level. The dataset of RNA-seq was deposited at DDBJ Sequence Read Archive (DRA) under the accession DRA009542.

## Results

### Imaging analysis of *A. oryzae* penetration into steamed rice

**Fig. 1:**
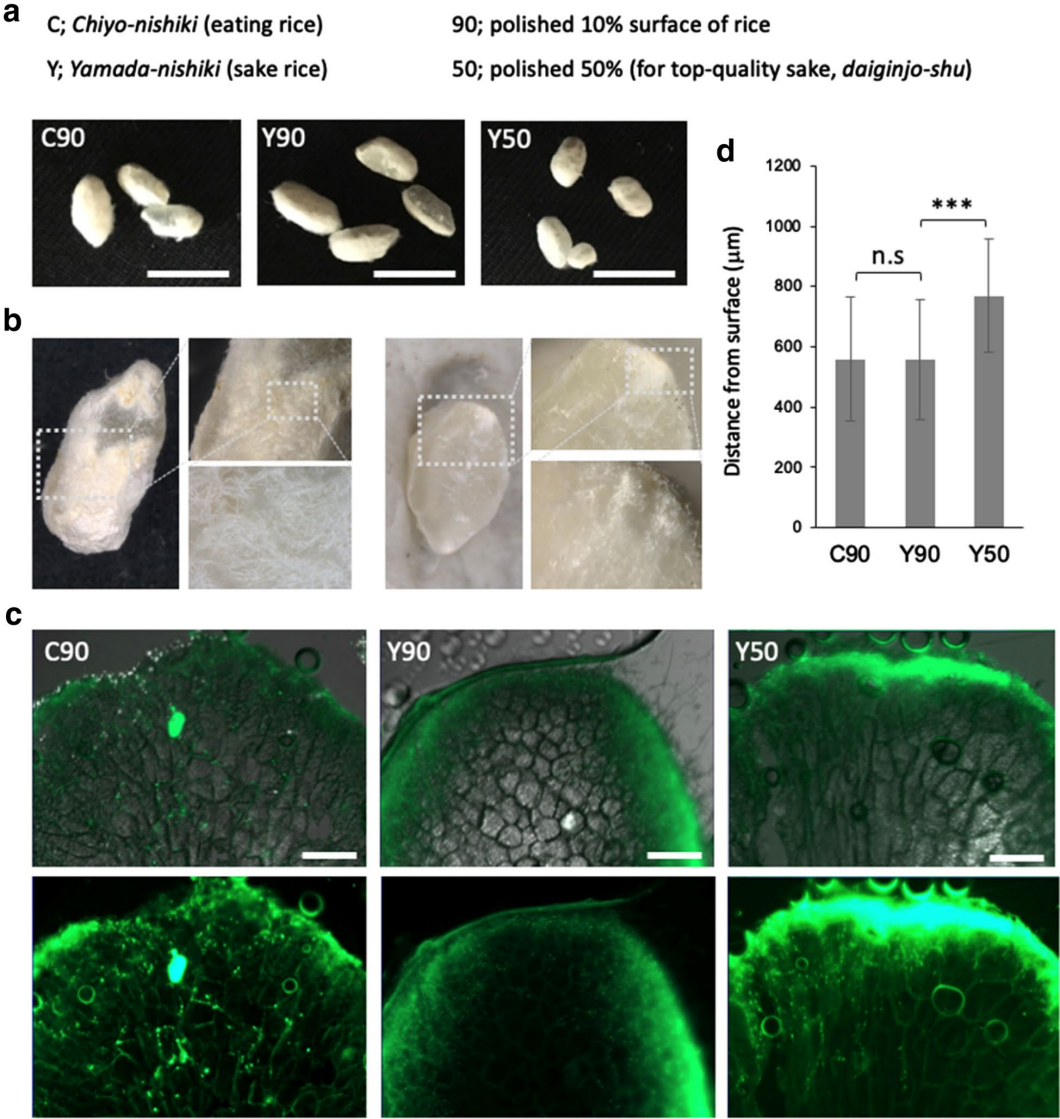
Imaging analysis of *A. oryzae* penetration into steamed rice. **a***Koji* of different rice races and polishing rates. *Chiyo*-*nishiki* (eating rice) and *Yamada*-*nishiki* (sake rice) polished 90% or 50%. Scale bar: 1 cm. **b** Images of section of *koji* by zoom microscopy. **c** Fluorescent images of *A. oryzae* (H2B-GFP) penetration into the steamed rice. The sections were sliced by cryo-microtome. Scale bars: 200 μm. **d** Distance of fungal penetration from surface in C90, Y90 and Y50. Error bar: S.D., n = 20 hyphae in 3 independent *koji*. ***P ≤ 0.001

*Yamada*-*nishiki* (sake rice) and *Chiyo*-*nishiki* (eating rice) were polished to 50% or 90% (removed 10% outside), and used to make *koji*, the steamed rice with *A. oryzae* mycelia (see “Materials and methods” section), referred as Y90, C90 and C50, respectively (Fig. 1a). *A. oryzae* mycelia grew on and in the *koji* pellets. We observed surfaces and cross sections of *koji* pellets by zoom microscopy (Fig. 1a, b). To evaluate the degree of invasive growth into the steamed rice, called ‘*haze*-*komi*’, we used an *A. oryzae* strain in which histone H2B is fused with GFP, to make *koji*. The *koji* was sliced in 30 μm sections using a cryomicrotome. The sections were observed by fluorescent microscopy. High intensity of GFP signals covered the periphery of rice in the Y90, C90 and C50 *koji* (Fig. 1c). Moreover, we could detect each hypha with GFP signal at the cellular level in the rice. We quantified the *haze*-*komi* by measuring how far hyphae penetrated from the *koji* surface in the Y90, C90 and C50 (Fig. 1d, Additional file 1: Figure S1A). The hyphal lengths from the *koji* surface in C90 and Y90 were comparable, 371 ± 60 and 311 ± 38 μm, respectively (n = 20 hyphae in 3 independent *koji*). Notably, the hyphae in Y50 penetrated more deeply 1.4–1.6 times, 501 ± 66 μm (n = 20 hyphae in 3 independent *koji*). There was no clear difference in the GFP intensities on the surface between the different types of rice.

### *A. oryzae* penetration inter-rice cells and intra-rice cells

**Fig. 2:**
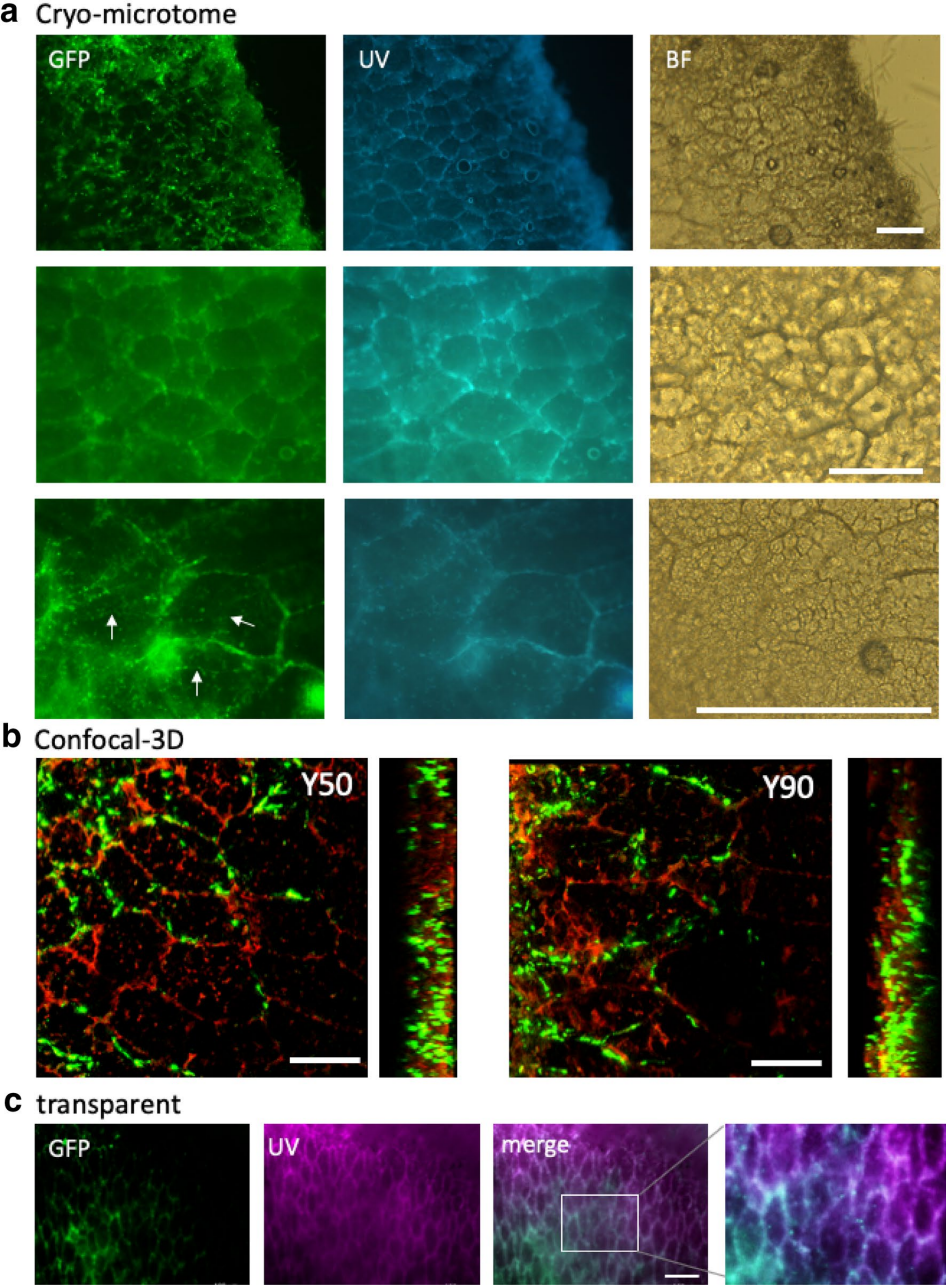
Fluorescent imaging analysis of *A. oryzae* penetration inter-rice cells and intra-rice cells. **a** Fluorescent images of *A. oryzae* (H2B-GFP) penetration inter-rice cells and intra-rice cells in Y50. The rice cells were shown by UV and BF (bright field). The sections were sliced by cryo-microtome. Hyphal growth inside of rice cells was indicated by arrows. Scale bars: 50 μm. **b** Confocal-3D imaging of the *koji* section from Y50 and Y90. *A. oryzae* (green), rice cells visualized by the autofluorescence (red). See also Additional files 2, 3: Movies S1 and S2. Scale bars: 50 μm. **c** The *koji* treated with the transparent reagent were imaged by fluorescent microscopy. *A. oryzae* (green), rice cells (purple). Scale bar: 100 μm

In the *koji* sections, rice endosperm cells were observed in bright field and UV light irradiation as well due to the autofluorescence (Fig. 2a). GFP signal from *A. oryzae* often indicated similar patterns with rice cells, suggesting that hyphae often grow between rice cells, which is consistent with the previous report [34]. In addition, higher magnification images showed hyphal growth inside of rice cells (Fig. 2a arrows, Additional file 1: Figure S1B). Confocal microscopy imaging of the *koji* sections confirmed that hyphae grew inside of rice cells and frequent co-localization of fungal signal on outlines of rice cells (Fig. 2b, Additional file 1: Figure S1C, Additional files 2, 3: Movie S1 and S2). The hyphal growth inter-rice cells and intra-rice cells were observed similarly in the Y90, C90 and C50.

We tested the chemical reagent TOMEI (see Materials and methods), that turns plant tissues transparent, for the *koji*. Confocal imaging visualized network-like GFP signal from *A. oryzae* co-localized with the arrangement of rice cells (Fig. 2c).

### SEM analysis of *A. oryzae* penetration intra-rice cells

**Fig. 3:**
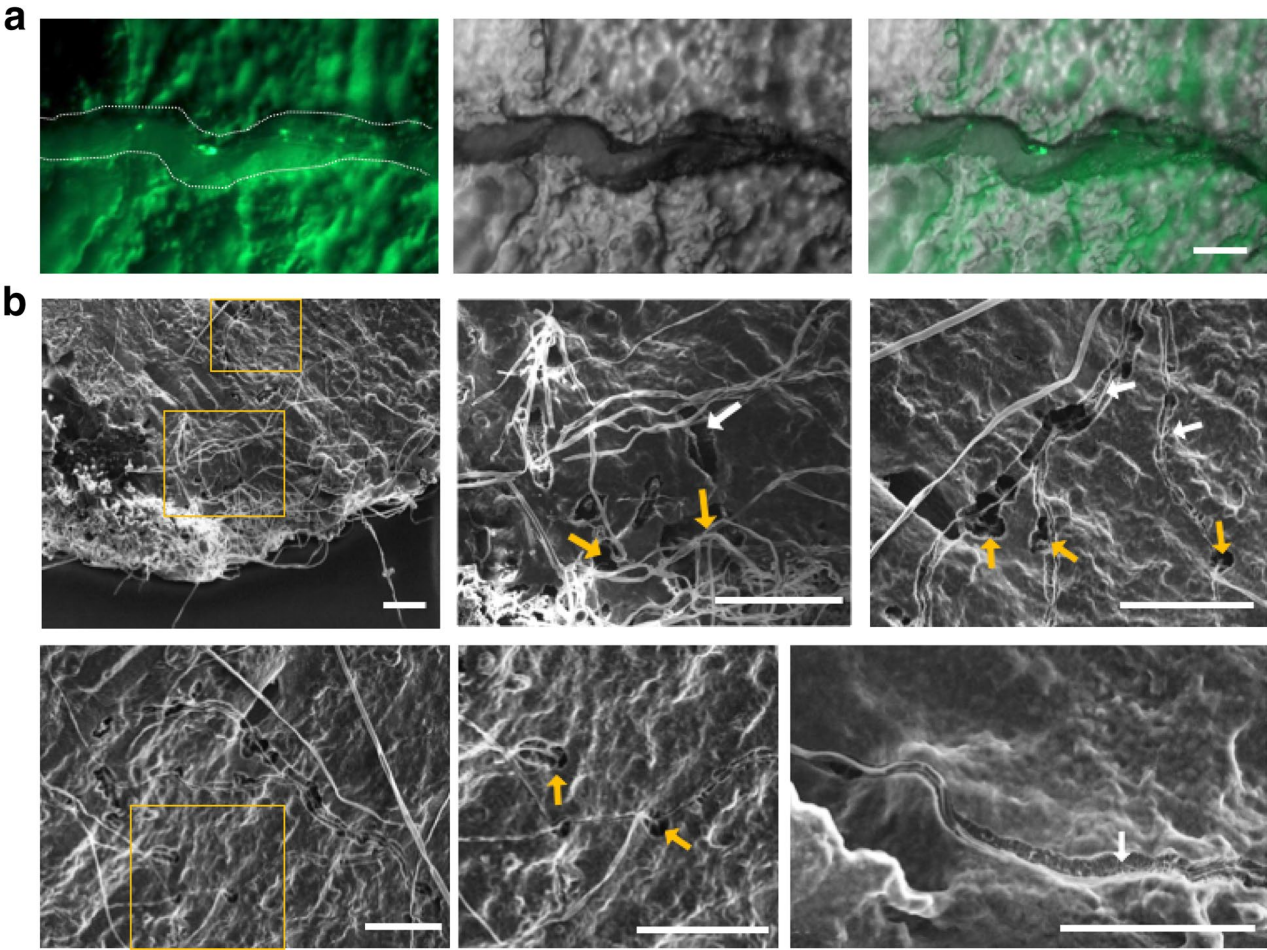
Fluorescent and SEM imaging analyses of *A. oryzae* penetration intra-rice cells. **a** Fluorescent images of *A. oryzae* (H2B-GFP) penetration intra-steamed rice Y50. Hyphal growth through surrounding space like a furrow in the rice (arrow and dotted line). Scale bars: 20 μm. **b** SEM images of the *koji* section. Scale bars: 100 μm. Cross sections of hyphae from or to holes (yellow arrows). Vertical sections of hyphae surrounding space like a furrow (white arrows)

We observed the cross sections of *koji* by fluorescent microscopy and found that some hyphae grew through surrounding space like a furrow in the rice (Fig. 3a, arrow and dotted line). Scanning Electron Microscopy (SEM) also indicated the hyphae in furrows on the rice cross sections (Fig. 3b, white arrows), although the shapes of rice cells were not clearly observed. The SEM imaging showed that some hyphae came from or went into a hole on the rice cross sections (Fig. 3b, yellow arrows). The holes and furrows appeared to be tunnels formed by the sugar degradation during the hyphal growth in rice cells, which is in agreement with the previous report [35].

### Time-lapse imaging of *A. oryzae* penetration into steamed rice

**Fig. 4:**
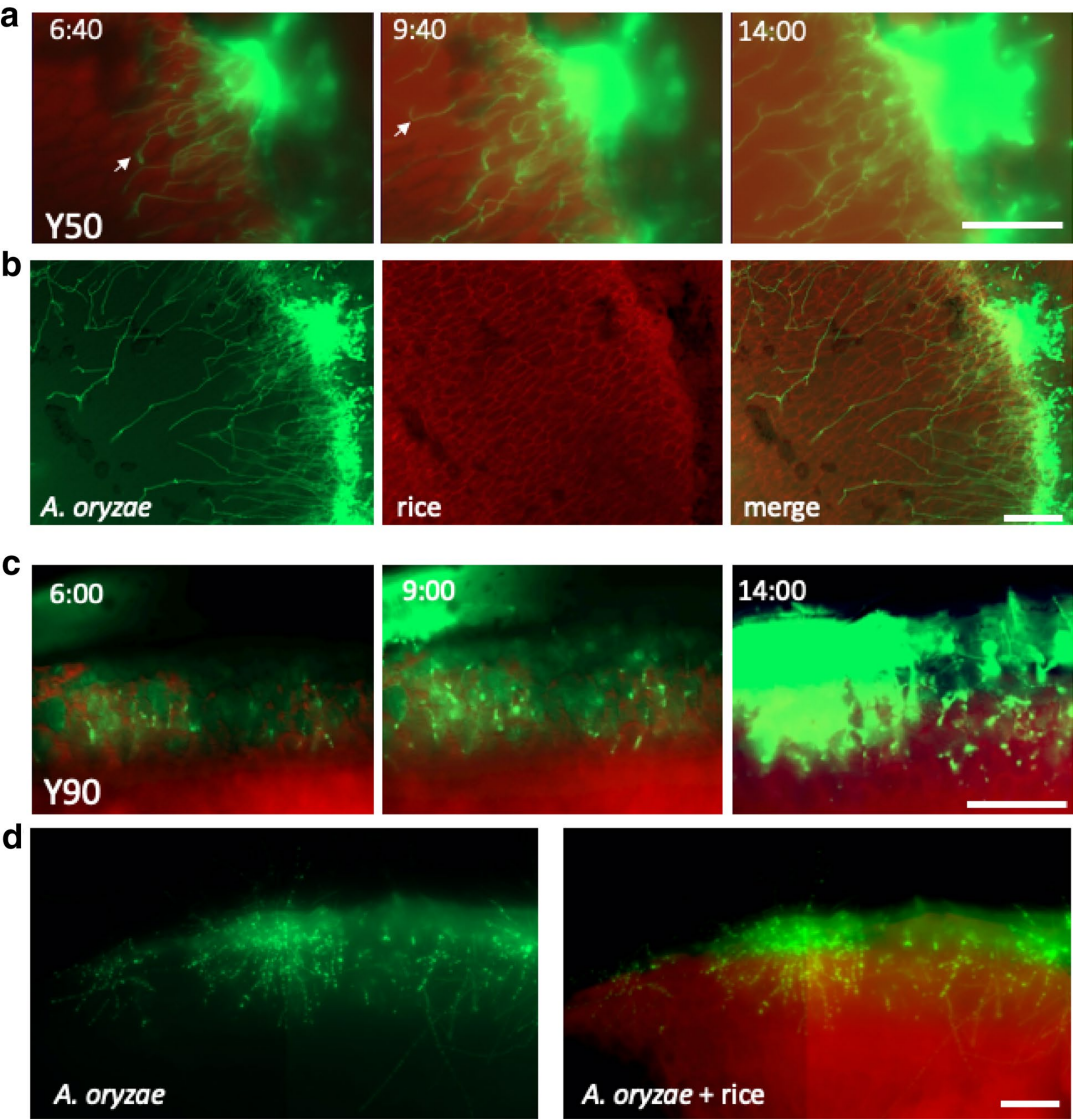
Time-lapse imaging analysis of *A. oryzae* penetration into steamed rice. The sections of *koji* Y50 (**a**, **b**) and Y90 (**c**, **d**), *A. oryzae* (green), rice cells (red), were imaged every 10 or 20 min for 14 h by fluorescent microscopy. See also Additional files 4, 5: Movies S3 and S4. Time-lapse images (**a**, **b**) and broader images at 14 h (**b**, **d**). **a** Some hyphae changed the growth direction after hitting the rice cell shape (arrows). Scale bars: 200 μm

To monitor the time course of *A. oryzae* growth into the steamed rice, we applied fluorescent live imaging for the cross sections of *koji* Y50 and Y90 (Fig. 4). The conidia were inoculated on the surface of rice, then the *koji* was incubated for 7 h. Z-stack images of the cross sections were taken every 10 or 20 min for 14 h. The Z-stack merged images were shown by time-lapse movies (Additional files 4, 5: Movies S3, S4). We could visualize *A. oryzae* hyphae (green) penetration into the steamed rice (red) (Fig. 4). In Y50, hyphae grew from the surface of rice towards the center of rice (Fig. 4a, b). While some hyphae grew through the rice cell shape, others changed the growth direction when they bumped against rice cells (arrows). The hyphae appeared to grow on the outside rice surface following the growth into the rice. In Y90, hyphal signal increased under the surface of rice (Fig. 4c, d). In contrast to Y50, most hyphae did not continue to penetrate towards the center of rice but grew close to the surface, mainly ~ 300 μm, with more branching than in Y50.

### X-ray CT analysis of *A. oryzae* penetration into steamed rice

**Fig. 5:**
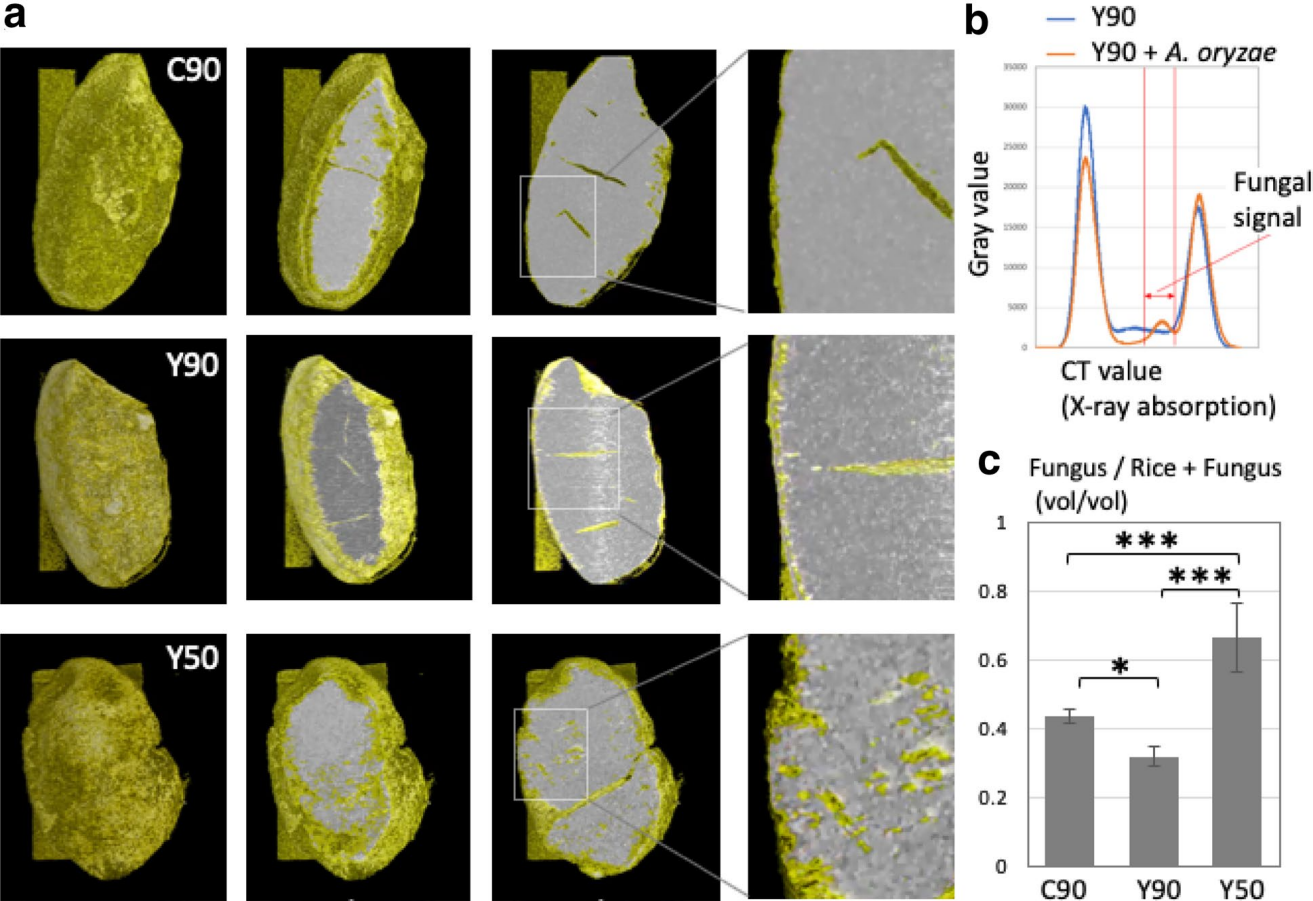
X-ray CT analysis of *A. oryzae* penetration into steamed rice. **a** Image sequence of X-ray CT analysis in C90, Y90 and Y50. See also Additional files 6, 7, 8: Movies S5, S6, S7. The rice and fungus are shown in white and yellow, respectively. **b** The fungal signals were determined by the peak in CT value (X-ray absorption) only found in Y90 + *A. oryzae*. **d** Fungal volume was calculated from the 3D data. Error bar: S.D., n = 3. * P ≤ 0.05, ***P ≤ 0.001

To complement fluorescence microscopy results and obtain more accurate information on mycelial penetration into the steamed rice, we performed a X-ray CT (Computed Tomography) scan analysis. The intact C90, Y90 and Y50 incubated for 48 h were set in the X-ray CT device. The X-ray CT scan produces cross-sectional tomographic images by use of computer-processed combinations of many X-ray measurements taken from different angles, allowing to observe the inside of objects without cutting. The 3D section images were shown by sequence images (Fig. 5a). The fungal signals were determined by the different peak found in CT value (X-ray absorption) line profiles between the *koji* (rice + *A. oryzae*) and the rice without the fungus (Fig. 5b). The rice and fungal mycelia are shown in white and yellow, respectively (Fig. 5a, Additional files 6, 7, 8: Movies S5, S6, S7). In the C90 and Y90, the fungal signals were detected mainly close to the surface of rice. In the Y50, in contrast, the signals were detected both close to the surface and inside of rice.

The rice and fungal volumes were calculated from the 3D data. The ratios of fungal volume per *koji*, rice + fungus, volumes were indicated in C90, Y90 and Y50 (Fig. 5c). The fungal ratios in the C90 and Y90 were 0.44 ± 0.02, 0.32 ± 0.03, respectively, while the fungal ratio in the Y50 was 0.67 ± 0.1 and significantly higher than those in the C90 and Y90, (n = 3, p ≤ 0.001). The X-ray CT scan analysis also supports deeper invasive growth in the Y50 than the C90 and Y90.

### Transcriptome analysis

We compared the transcriptome profiles of *A. oryzae* in Y50, Y90 and C90 by RNA-seq analysis. The effects of the variety of rice and the polishing rate on the growth of *A. oryzae*, enzyme production, and metabolism production in *sake*-*koji* have been investigated previously [36]. One of the most important roles of *A. oryzae* in rice *koji* is the supply of enzymes, vitamins, nutrition, such as glucose, amino acids and peptides, that are necessary for sake brewing. From this viewpoint, we compared the expression of genes related to these processes (Additional file 9: Table S1). A comparison between Y50 and Y90 is summarized in Table 1. Digestion of starch and supply of glucose by amylases are the basis of alcohol fermentation. The expression of genes for α-amylases (*amyA*, *amyB* and *amyC*) and glucoamylase (*glaA*) was 5.2 and 1.7 times higher in Y50 than in Y90, whereas the expression level of maltases, which hydrolyse maltose to glucose, was comparable. The metabolic genes in glycolysis, TCA cycle and electron transport chain were compared by a heatmap (Additional file 10: Figure S2).

**Table 1:** Comparison of the expression of genes between Y50 and Y90

Function	Gene	Y50/Y90
α-Amylase, glucoamylase	*amyA*-*C*, *glaA*	5.2, 1.7
Acid protease	*pepA*	0.08
Fatty acid synthase	*fasA*, *fasB*	5.6, 7.4
Delta-9-stearic acid desaturase	*sdeA*, *sdeB*	13.7, 20.2
Phytase	*phyA*	4.4
Acid phosphatase	*aphA*, *pacA*, *phoA*	2.0, 1.6, 1.6
Alkaline phosphatase	*pho8*	5.8
Thiamine synthesis	*thiA*, *thi6*	4.9, 3.9
Biotin synthesis	*bioF*, *bioA*	6.9, 3.2
Pantotate synthesis	*apbA*, *panB*	4.9, 3.0
Conidiophore development, solid state culture regulation	*flbA*, *flbB*, *flbD*, *brlA*, *flbC*	8.0, 1.6, 2.5, 2.4, 4.7

Acid proteases are involved in the digestion of the main protein of rice glutelin, also called oryzanin. The enzyme breaks down the protein body containing glutelin, resulting in disruption of the rice structure. Carboxypeptidases degrade peptides and supply amino acids. The expression of major acid protease gene, *pepA*, was 13 times lower in Y50 than that in Y90, whereas the expression level of carboxypeptidase genes was almost unchanged.

The outer surface of rice, the aleurone layer, is rich in lipids and fatty acids, and their contents decreases as the polishing rate increases [37]. When the polishing rate decreases, the ratio of saturated fatty acids and unsaturated fatty acids changes [37]. In sake brewing, fatty acids are important in the production of yeast-derived aroma components (*ginjo* aroma, especially ethyl caproate). When the unsaturated fatty acid content increases, the production of ethyl caproate in yeast is suppressed. The secreted lipase is necessary for supplying lipids and fatty acids to yeast. The expression of fatty acid synthase genes, *fasA* and *fasB*, was 5–7 times higher in Y50 than those in Y90. Additionally, the expression of *sdeA* and *sdeB* genes for delta-9-stearic acid desaturase, which converts palmitic acid and stearic acid to palmitoleic acid and oleic acid, respectively, was 13–20 times higher in Y50 than Y90, whereas the expression level of lipase genes was almost unchanged.

Supply of phosphate affects the following yeast fermentation [38]. Phytic acid is a preserved state of phosphate in plants, and its content decreases as the rice polishing rate increases [37]. Phytases function to release phosphate from phytic acid [39]. The expression of phytases, acid phosphatases and alkaline phosphatases tend to increase in Y50 compared to Y90.

Most of sake yeasts lack some of the genes for vitamin biosynthesis. In addition, enzymes involved in fermentation require vitamins as cofactors. Supply of vitamins from *koji* is essential to proceed with fermentation [40]. Vitamins are abundant in the outer surface layer and germ of rice, and their amounts decrease as the rice polishing rate increases [41]. The expression of synthesis genes for thiamine, pantothenate and biotin (Vitamin B1, B5 and B7, respectively) increased in Y50 compared to Y90.

Beside the genes related to brewing and fermentation, genes for conidiophore development, *flbA*-*D* and *brlA* [42], were up-regulated in Y50 compared to Y90, whereas *abaA*, which is required for phialide differentiation, was unchanged. In *A. oryzae*, *flbC* was reported to regulate the expression of genes specifically under solid-state cultivation conditions, possibly independent of the conidiation regulatory network [43].

### Increase number of nuclei in *A. oryzae* hyphae

**Fig. 6:**
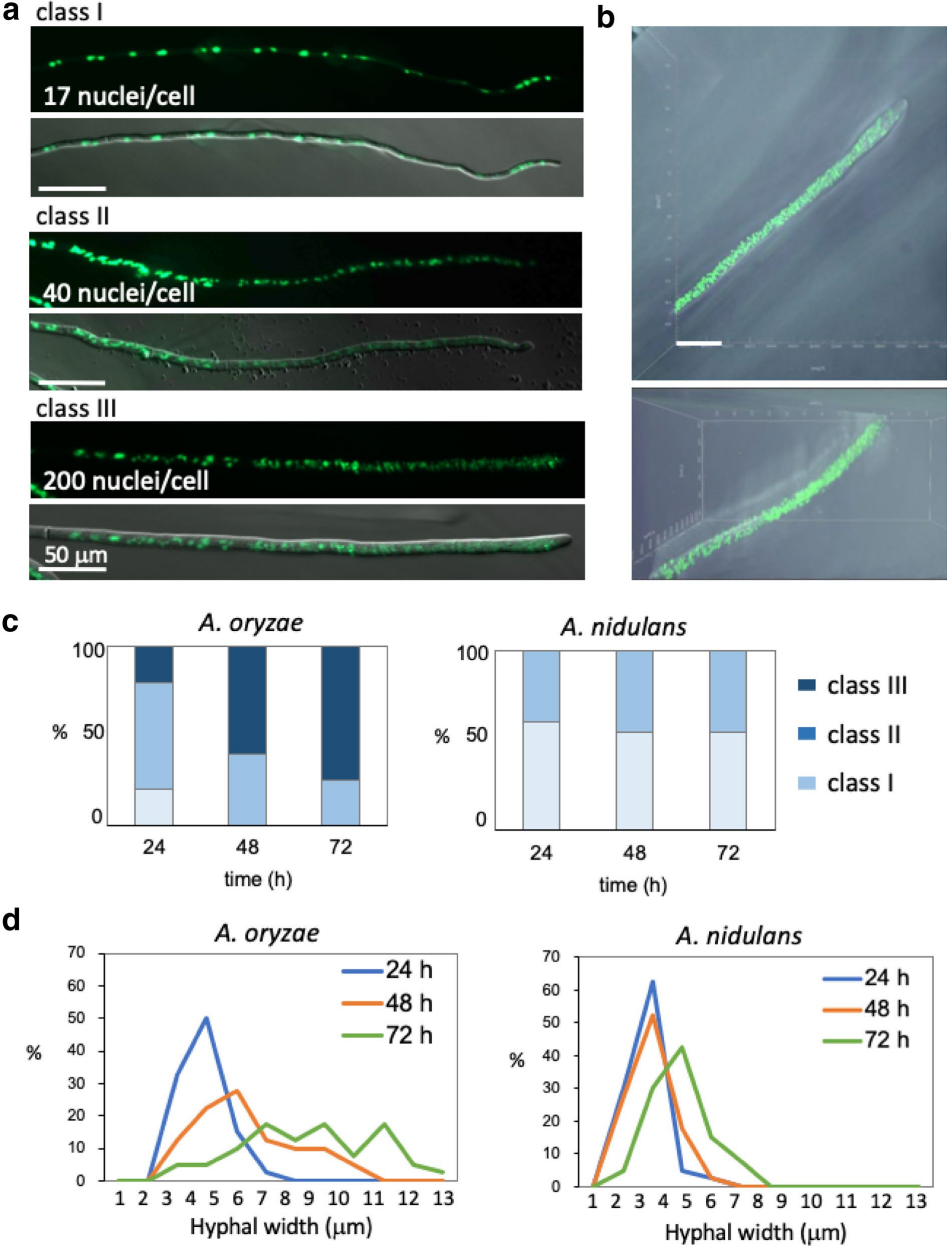
Increase of nuclear number in *A. oryzae* but not *in A. nidulans.***a** Images of nuclei in the tip compartment of *A. oryzae*. The septal positions are shown by arrows. Scale bars: 50 μm. **b** Confocal-3D imaging of nuclei in the *A. oryzae* tip compartment. See also Additional file 12: Movie S8. Scale bar: 40 μm. **c** Ratio of nuclear distribution pattern classified as class I, II, III (**a**) in *A. oryzae* (left) and *A. nidulans* (right) at 24-, 48- and 72-h growth (n = 20). **d** Ratio of hyphal width in *A. oryzae* (left) and *A. nidulans* (right) at 24-, 48- and 72-h growth (n = 100)

We observed nuclei labeled with GFP of *A. oryzae* in the *koji* and found that the number of nuclei often varied in each hypha (Additional file 11: Figure S3). Since the increase of nuclei in *A. oryzae* was predicted to be correlated to the high secretion capacity of several enzymes, we focused on the phenotype. The nuclear distribution in *A. oryzae* has been analyzed in hyphae and especially in conidia [44, 45], which indicated multi-nuclear conidia; the number of nuclei in each conidium varied from 1 to 7 in *A. oryzae* strains used in sake brewing. We investigated the nuclear distribution in *A. oryzae* hyphae grown in detail by using the minimal medium but not the rice *koji*. Some of hyphae contained less than 20 nuclei in the tip compartments, the hyphal cell from the tip to the first septum (Fig. 6a, upper). Other hyphae contained more than 200 nuclei in the tip compartments (Fig. 6a, lower). The hypha containing such a high number of nuclei was imaged by the Z-stack confocal microscopy and shown in 3D imaging (Fig. 6b, Additional file 12: Movie S8).

We classified the pattern of nuclear distribution into three types as follows. Class I; nuclei distribute at a constant interval without overlapping. Class II; nuclei align but sometimes overlap. Class III; nuclei scattered throughout hyphae but not aligned. We counted the ratios of class I–III in the time course at 24-, 48- and 72-h growth (Fig. 6c). At 24 h, class II was large, while class I and III were approximately 20%. At 48 and 72 h, class III increased to 60% and more than 70%, respectively. The class III hyphae were usually thicker than those of class I. We measured the hyphal width at tip compartments at 24-, 48- and 72-h of growth (Fig. 6d). At 24 h, the hyphal width was 3 to 6 μm. At 48 h, the ratio of 7 to 10-μm hyphae increased, then at 72 h, the hyphal width ranged 3 to 12 μm.

As a comparison, we investigated the nuclear distribution in the model fungus *Aspergillus nidulans* as well in the same way. The ratios of class I and II did not vary approximately 40% and 60%, respectively, in the time course at 24-, 48- and 72-h (Fig. 6c). The hyphal widths were comparable at 24 and 48 h, then the peak shifted by 2 μm wider at 72 h (Fig. 6d). These results indicate that *A. oryzae* increases the nuclear number and hyphal width in the time course of 1–3 days, which may correlate with the high secretory capacity of several enzymes.

### Synchronous mitosis in *A. oryzae*

**Fig. 7:**
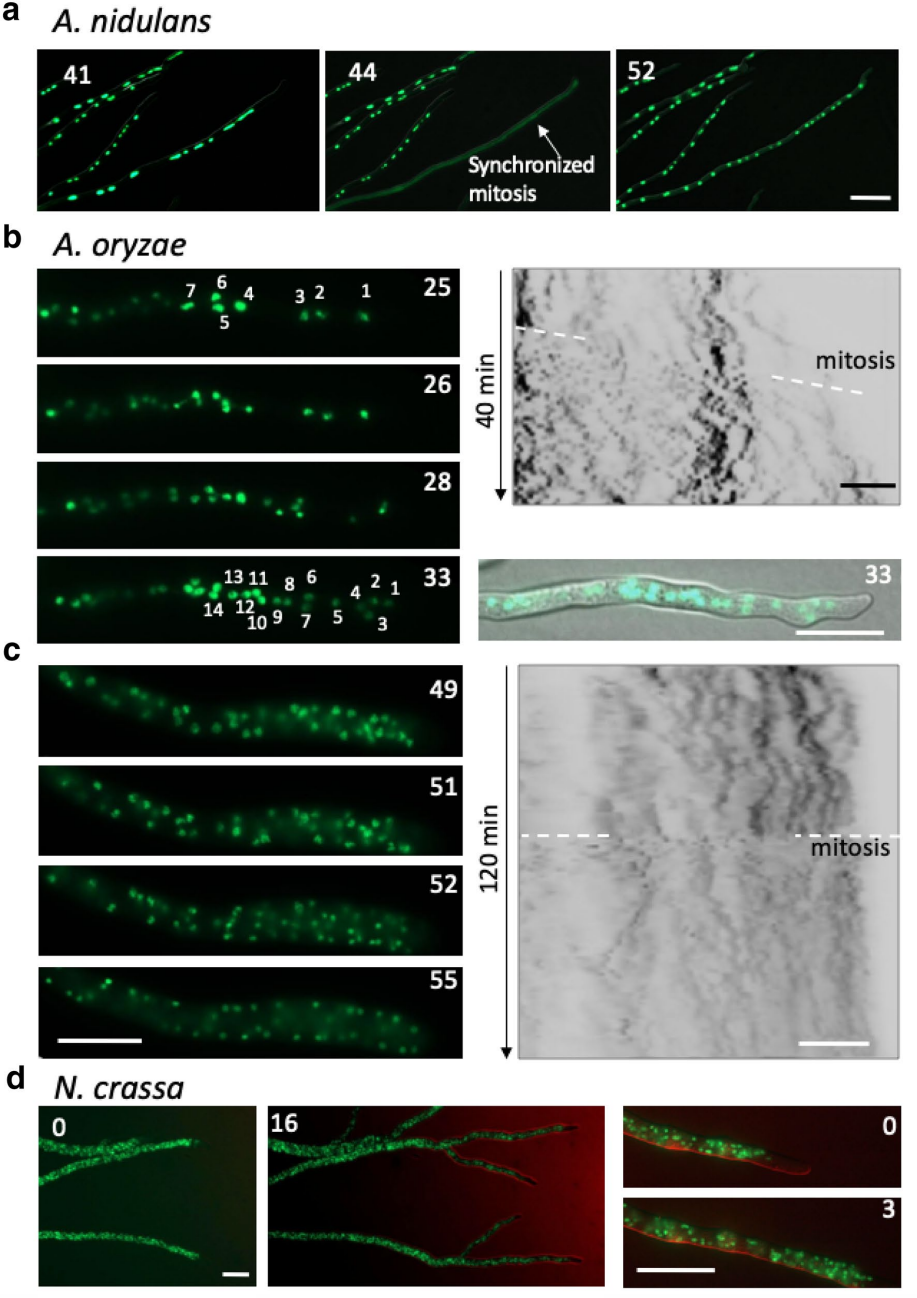
Nuclear distribution and mitosis in *A. nidulans*, *A. oryzae* and *N. crassa.***a** Image sequence of synchronized mitosis in *A. nidulans* observed every minute for 3 h by fluorescent microscopy. See also Additional file 13: Movie S9. **b**, **c** Image sequence of nuclei in the *A. oryzae* tip compartment from 1 day incubation (Additional file 14: Movie S10) and 3 days incubation (Additional file 15: Movie S11). Kymographs of nuclear distribution along the hypha. **d** Image sequence of nuclei in *N. crassa* from Additional files 16, 17: Movies S12, S13. The elapsed time is given in minutes (**a**–**d**). **a**–**c** Scale bars: 20 μm, (**d**) Scale bars: 50 μm

To analyze the mechanism of the increase in nuclear number, we investigated the nuclear distribution in mitosis. Synchronous nuclear division in a hyphal compartment has been known in *A. nidulans* [32, 46, 47]. We used the *A. nidulans* strain expressing the nuclear localizing signal of the transcription factor StuA tagged with GFP [32, 48]. The GFP protein localizes in nuclei in interphase, while they move out from the nuclei to cytoplasm due to partial disassembly of nuclear pore complex during closed mitosis (Fig. 7a, Additional file 13: Movie S9) [49, 50]. The nuclear membrane envelope is intact but permeable, known as partially open mitosis. After mitosis, GFP signals moved back in the two-fold number of nuclei.

We investigated the nuclear distribution in *A. oryzae* mitosis, where histone H2B labeled with GFP remained in nuclei during mitosis. Time-lapse imaging revealed the synchronized mitosis within 5 min in the tip compartment (Fig. 7b, Additional file 14: Movie S10). Even in the class III hypha, a lot of nuclei divided within 5 min in the tip compartment (Fig. 7c, Additional file 15: Movie 11). We performed Z-stack and time-lapse of the class III hyphal mitosis and revealed the synchronized mitosis, although the time and space resolutions were not sufficient to demonstrate all nuclei enter mitosis at same time, Additional file 15: Movie S11 showing that the H2B-GFP signals undergo condensation simultaneously suggests a synchronous nuclear division. The nuclei moved a lot after mitosis in *A. oryzae* which is consistent with that in *A. nidulans* [49]. Since the size of the H2B-GFP signal did not show significant difference from 24 to 72 h, 2.5 ± 0.3 and 2.5 ± 0.2 μm (n = 10 from Additional files 14, 15: Movies S10 and S11), the increase in nuclear number is not due to fragmentation of nuclei.

The nuclear distribution in class III hypha of *A. oryzae* resembles that of the another model fungus *Neurospora crassa* (Fig. 7d, Additional files 16, 17: Movies S12, S13), whose mitosis is not clear to be synchronous or not [51].

## Discussion

We improved the base of imaging analyses to evaluate ‘*haze*-*komi*’, fungal penetration into steamed rice, by using fluorescent microscopy. Our analyses indicate that *A. oryzae* hyphae grew more deeply into 50% polished rice Y50 than 90% polished rice Y90 and C90. Since proteins and lipids are abundant near the surface in a grain of rice [27], the 50% polished rice consists of mainly starch. It is likely that *A. oryzae* mycelia grow near the surface in 90% polished rice due to sufficient supply of nutrients near the surface, while the mycelia grow deeply into 50% polished rice to search for nutrients and water as well. That is believed by experience of sake brewers as evaluated by several methods [22–25], which correlates with our results. We visualized fungal hyphae in cellular level in rice *koji*. Our data support that hyphae penetrate between rice cells and grow inside of rice cells. These imaging analyses are widely applicable by proper staining for rice *koji* using different *A. oryzae* strains, different rice races and polished rates. These approaches contribute to monitoring the status and quality of rice *koji* and to screening the combination of *A. oryzae* strains and rice races according to favored qualities of sake.

Another important finding is that *A. oryzae* increases the nuclear number drastically, 20 to more than 200 nuclei in the hyphal tip compartment. The increase of nuclear number is correlated with the hyphal width. The hyphal growth did not slow down at 72 h, in addition septation sites were usual. The hyphal tip compartments contain more nuclei at higher density. That phenomenon was not observed in *A. nidulans* (Fig. 6) and has not been reported in other filamentous fungi. One of the reasons that could explain the increase of nuclear number was that mitosis is not synchronized, resulting in gradual increase of nuclear number. Asynchronous nuclear division cycles are known in *Ashbya gossypii* [52], which is a filamentous fungus closely related to the budding yeast *S. cerevisiae*. We found, however, that a lot of nuclei even in the class III hypha of *A. oryzae* divided synchronously (Fig. 7b, c). Another possibility is a defect in cell cycle checkpoint at G1/S transition [53]. Cellular size is usually maintained by the checkpoint at G1/S transition of cell cycle, which represses the mitosis until the cell grows to a proper size. The cellular size in *A. oryzae* increased in the time course of 1–3 days, which might be caused by any defect in the G1/S checkpoint. That might allow the increase of nuclear number in the larger cell, however, the mechanism remains unknown. The increase of nuclear number in *A. oryzae* is likely correlated with the secretory capacity of several enzymes. The characteristic could be an extremely important feature of *A. oryzae.* Since the increase of nuclear number is not clearly observed in closely related species *A. flavus* (unpublished data), the characteristic may be a selectively acquired character during a long history of brewing. Future research will explore the molecular mechanism of increased nuclear number in *A. oryzae* and possible link to increased secretory capacity.

## Conclusion

Our imaging analyses indicate that *A. oryzae* hyphae grew more deeply into 50% polished rice Y50 than 10% polished rice Y90 and C90. Another important finding is that *A. oryzae* increases the nuclear number drastically, 20 to more than 200 nuclei in the hyphal tip compartment. The increases of nuclear number may be a selectively acquired characteristic for the high secretory capacity that has occurred during the long history of cultivating *A. oryzae*.

## Supplementary information

**Additional file 1. Figure S1.** Expanded fluorescent images of A. oryzae penetration inter-rice cells and intra-rice cells.

**Additional file 2. Movie S1.** Confocal-3D movie of the koji Y50.

**Additional file 3. Movie S2.** Confocal-3D movie of the koji Y90.

**Additional file 4. Movie S3.** Time-lapse movie of koji Y50.

**Additional file 5. Movie S4.** Time-lapse movie of koji Y90.

**Additional file 6. Movie S5.** X-ray CT of C90.

**Additional file 7. Movie S6.** X-ray CT of Y90.

**Additional file 8. Movie S7.** X-ray CT of Y50.

**Additional file 9. Table S1.** Transcriptome analysis of genes related to sake brewing.

**Additional file 10. Figure S2.** Heatmap of gene expression in glycolysis, TCA cycle and electron transport chain.

**Additional file 11. Figure S3.** Images of nuclei in A. oryzae hyphae grown in koji.

**Additional file 12. Movie S8.** Confocal-3D movie of nuclei in the A. oryzae tip compartment.

**Additional file 13. Movie S9.** Time-lapse movie of synchronized mitosis in A. nidulans.

**Additional file 14. Movie S10.** Time-lapse movie of synchronized mitosis in A. oryzae at 1 day.

**Additional file 15. Movie S11.** Time-lapse movie of synchronized mitosis in A. oryzae at 3 days.

**Additional file 16. Movie S12.** Time-lapse movie of nuclear movement in N. crassa.

**Additional file 17. Movie S13.** Time-lapse movie of nuclear movement in N. crassa.

## Data Availability

The dataset of RNA-seq was deposited at DDBJ Sequence Read Archive (DRA) under the accession DRA009542.
