# Supplementary figures and images for: Invasive growth of Aspergillus oryzae in rice koji and increase of nuclear number
Source: Fungal Biol Biotechnol. 2020 Jun 5;7:8. doi: 10.1186/s40694-020-00099-9 (PMC7275602; doi:10.1186/s40694-020-00099-9)

Additional file 1: Figure S1

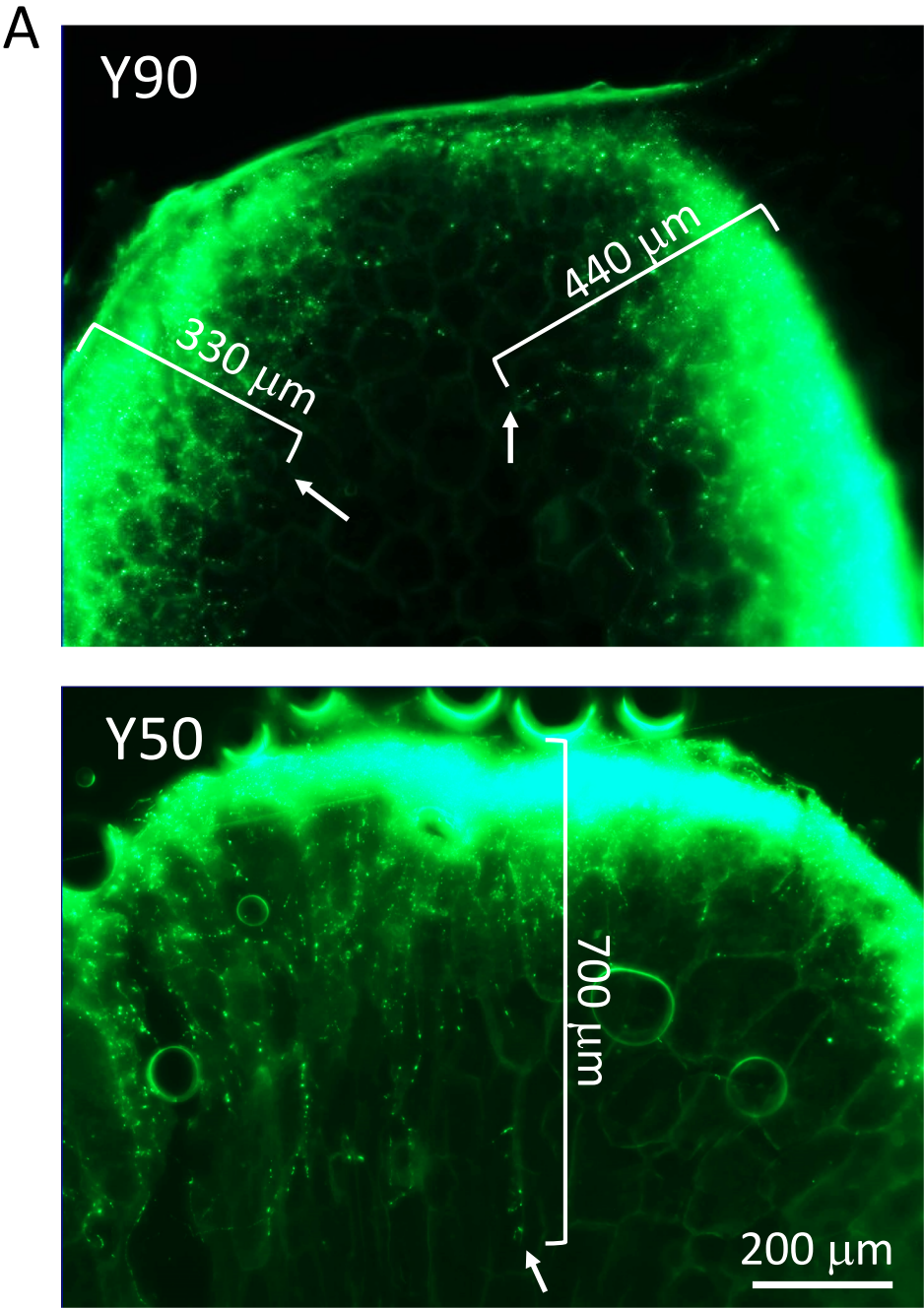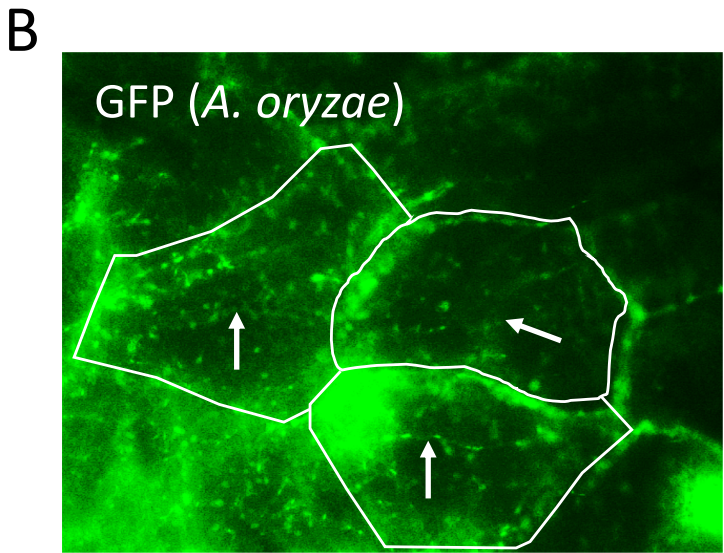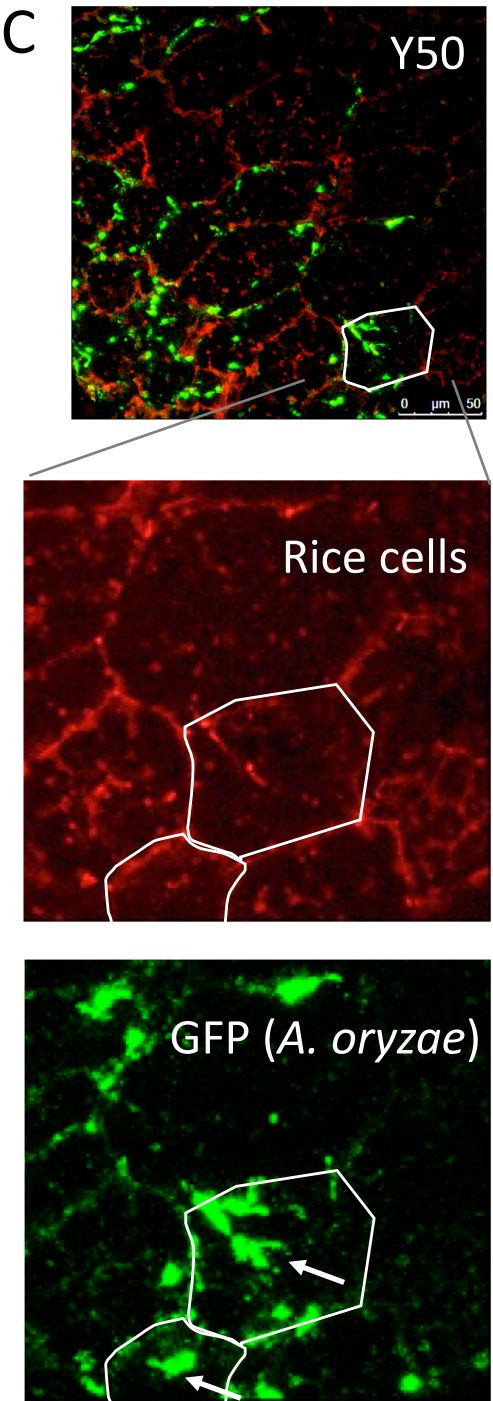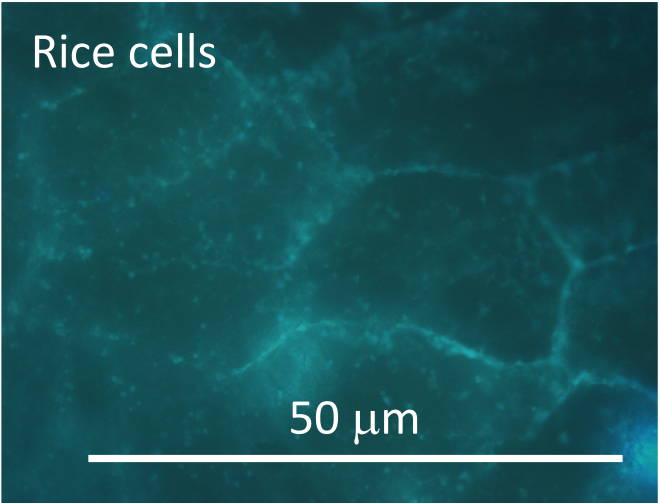

Supplement: Supplementary file 1 — Additional file 1. Figure S1. Expanded fluorescent images of A. oryzae penetration inter-rice cells and intra-rice cells. [file 40694_2020_99_MOESM1_ESM.pdf]

Additional file 10: Figure S2

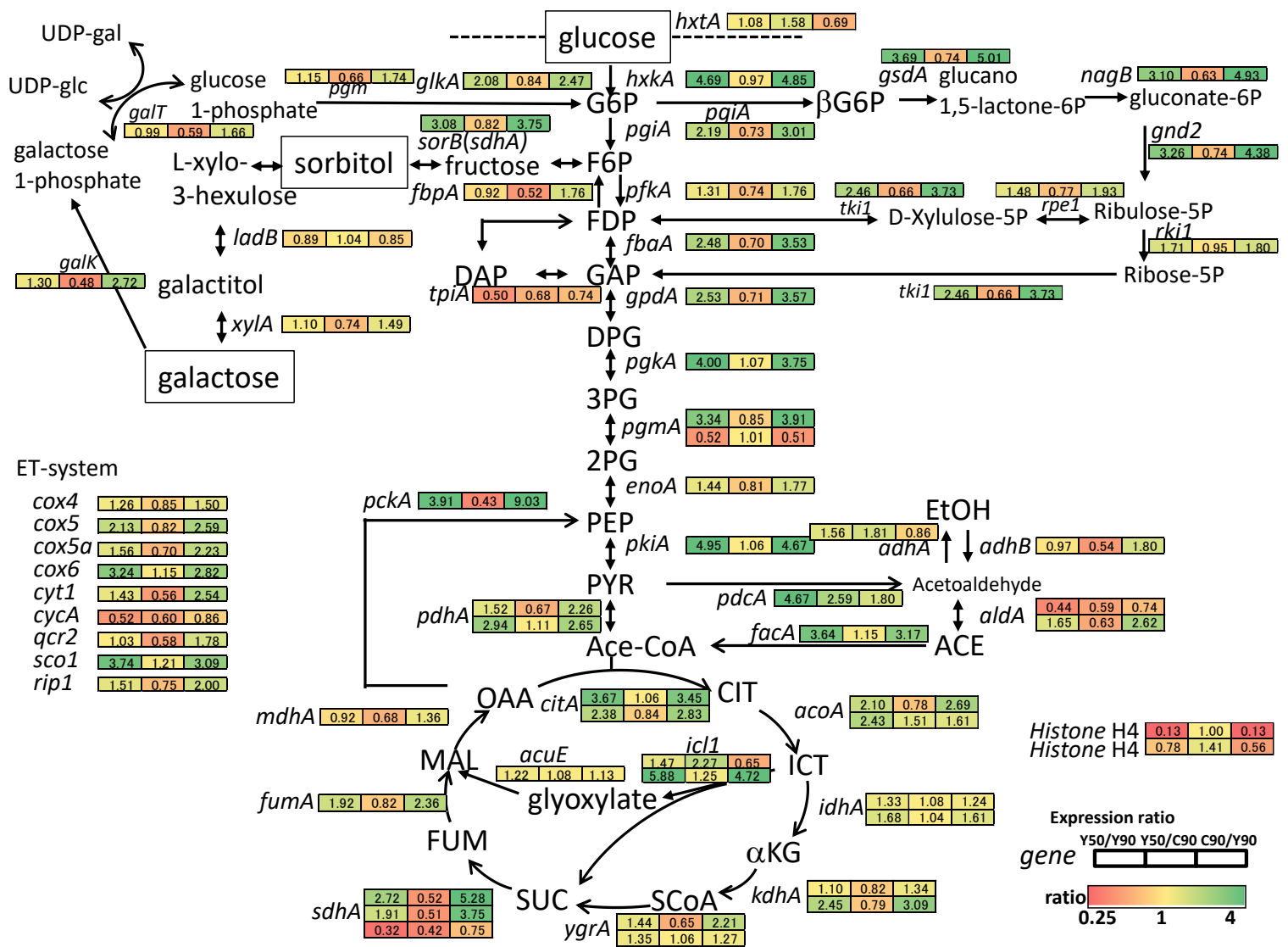

Supplement: Supplementary file 10 — Additional file 10. Figure S2. Heatmap of gene expression in glycolysis, TCA cycle and electron transport chain. [file 40694_2020_99_MOESM10_ESM.pdf]

Additional file 11: Figure S3

*A. oryzae* GFP-H2B in rice *koji*

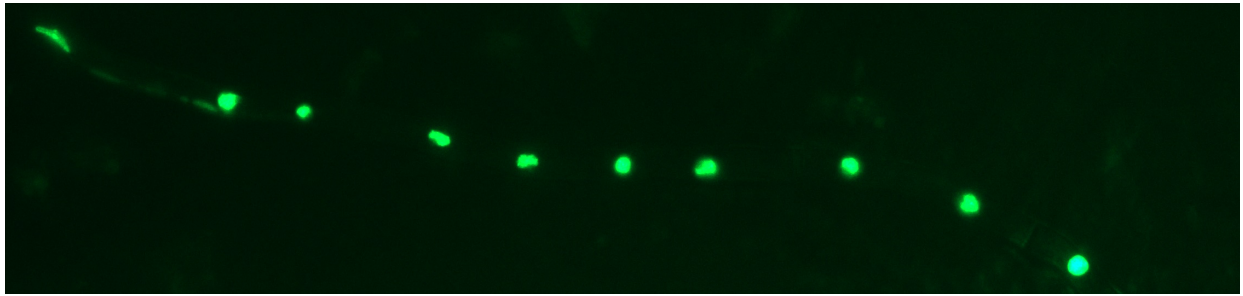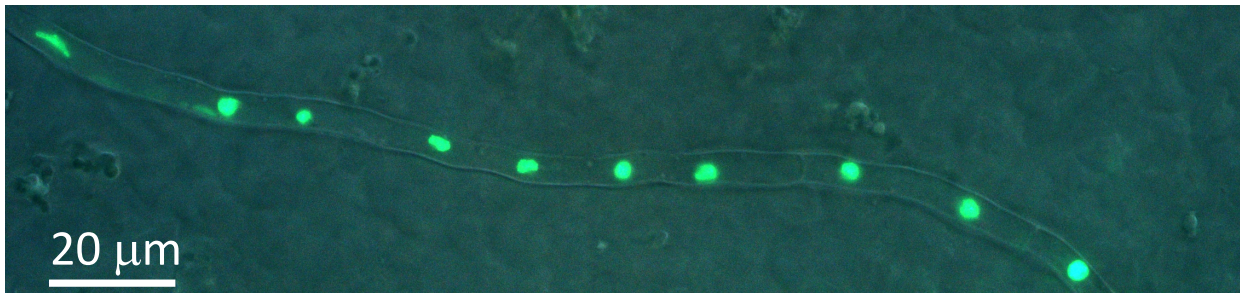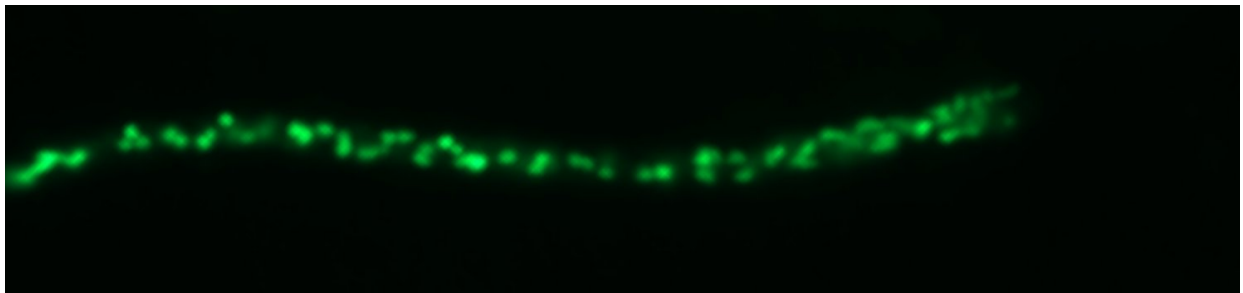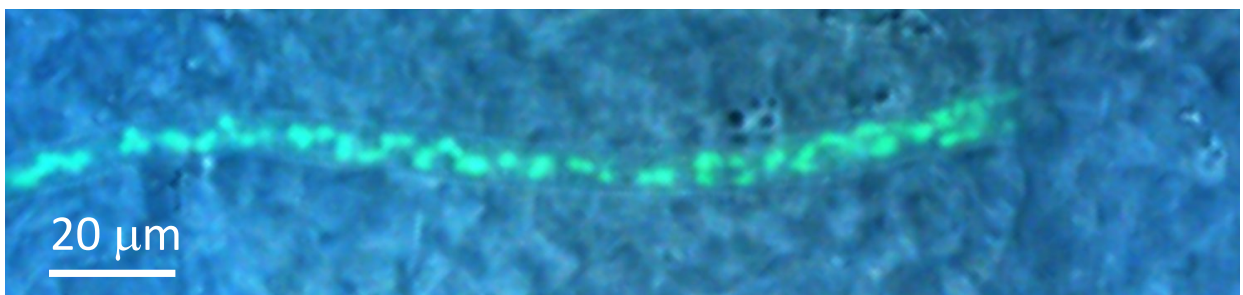

Supplement: Supplementary file 11 — Additional file 11. Figure S3. Images of nuclei in A. oryzae hyphae grown in koji. [file 40694_2020_99_MOESM11_ESM.pdf]
